# Splicing Characterization of *CLCNKB* Variants in Four Patients With Type III Bartter Syndrome

**DOI:** 10.3389/fgene.2020.00081

**Published:** 2020-02-21

**Authors:** Chunli Wang, Yuan Han, Jiaran Zhou, Bixia Zheng, Wei Zhou, Huaying Bao, Zhanjun Jia, Aihua Zhang, Songming Huang, Guixia Ding, Fei Zhao

**Affiliations:** ^1^ Nanjing Key Laboratory of Pediatrics, Children's Hospital of Nanjing Medical University, Nanjing, China; ^2^ Department of Nephrology, Children's Hospital of Nanjing Medical University, Nanjing, China; ^3^ Jiangsu Key Laboratory of Pediatrics, Nanjing Medical University, Nanjing, China

**Keywords:** classical Bartter syndrome, *CLCNKB*, synonymous variant, abnormal RNA splicing, hypokalemia

## Abstract

**Objective:**

Type III Bartter syndrome (BS) is caused by loss-of-function mutations in the gene encoding basolateral chloride channel ClC-Kb (*CLCNKB*), and is characterized by hypokalemic metabolic alkalosis and hyperreninemic hyperaldosteronism. Here, we investigated the molecular defects in four Chinese children with clinical manifestations of Bartter syndrome.

**Methods:**

The genomic DNA of the four patients was screened for gene variations using whole-exome sequencing (WES). The candidate variants were validated by direct Sanger sequencing. Quantitative PCR (qPCR) was subsequently performed to confirm the whole *CLCNK* gene deletion mutation. A minigene assay and reverse transcription PCR (RT-PCR) were performed to analyze the effect of splice variants *in vitro*.

**Results:**

Our patients showed early onset age with hyponatremia, hypokalemia, hypochloremia, repeated vomiting and growth retardation, suggesting Bartter syndrome. Genetic analysis revealed that all patients carried compound heterozygous or homozygous truncating variants in the *CLCNKB* gene. In particular, we identified a novel nonsense variant c.239G > A (p.(Trp80*)), two splice site variants (c.1053-1 G > A and c.1228-2A > G), a whole gene deletion, and a novel synonymous variant c.228A > C (p.(Arg76Arg)) which located -2 bp from the 5′ splice donor site in exon 3. Furthermore, our *in vitro* minigene analysis revealed c.228A > C, c.1053-1G > A, and c.1228-2A > G cause the skipping of exon 3, exon 12, and exon 13, respectively.

**Conclusion:**

Our results support that the whole *CLCNKB* gene deletion is the most common mutation in Chinese patients with type III BS, and truncating and whole gene deletion variants may account for a more severe phenotype of patients. We verified the pathogenic effect of three splicing variants (c.228A > C, c.1053-1G > A, and c.1228-2A > G) which disturbed the normal mRNA splicing, suggesting that splice variants play an important role in the molecular basis of type III BS, and careful molecular profiling of these patients will be essential for future effective personalized treatment options.

## Introduction

Bartter syndrome (BS) is an autosomal recessive inherited renal disorder characterized by renal salt wasting, hypokalemic metabolic alkalosis, elevated renin-aldosterone levels with normal-to-low blood pressure, hypercalciuria and normal serum magnesium levels (Bartter et al., 1962). In recent years, Bartter syndrome has been classified into five types (types I–V) based on the different underlying disease-causing genes *SLC12A1, KCNJ1, CLCNKB, BSND* and *MAGED2* (Seyberth, 2008; Al Shibli and Narchi, 2015; Laghmani et al., 2016).

Among them, BS type III (OMIM, #607364) is a highly heterogeneous presentation characterized by an onset in early childhood, hypocalciuria, or normocalciuria and nephrocalcinosis (Rodriguez-Soriano et al., 2005). BS type III is due to loss of function of the chloride channel protein ClC-Kb encoded by the *CLCNKB* gene (OMIM, #602023) (Simon et al., 1997). The ClC-Kb channel belongs to the voltage-dependent chloride channel (ClC) family, which has 12 transmembrane domains and intracellular amino and carboxy termini. ClC-Kb is expressed in the thick ascending limb of Henle's loop, distal tubule, and cortical collecting tubule, and predominantly mediates the tubular reabsorption of chloride in the kidney (Jentsch et al., 2002). Impaired ClC-Kb function reduces chloride and sodium reabsorption in the renal tubules, resulting in salt loss in urine (Naesens et al., 2004).

According to the HGMD (Human Gene Mutation Database; http://www.hgmd.cf.ac.uk), more than 152 mutations have been reported in the *CLCNKB* gene, including 86 missense mutations or nonsense mutations, 17 splice site mutations, 40 large and small deletions, 5 small insertions and 2 complex rearrangements.

In this study, we identified four patients with clinical manifestations of Bartter syndrome, which expands the spectrum of mutations of the *CLCNKB* gene in the Chinese population. The novel synonymous variant c.228A > C and two classical splice site variants (c.1053-1 G > A and c.1228-2A > G) were predicted to be deleterious in mRNA splicing. Our minigene assay verified the pathogenic effect of three splicing variants (c.228A > C, c.1053-1 G > A, and c.1228-2A > G) which disturbed the normal mRNA splicing *in vitro*, suggesting that a significant portion of synonymous and splice site variants play an important role in the molecular basis of type III BS.

## Materials and Methods

### Whole-Exome Sequencing

Genomic DNA was extracted from the peripheral blood of all participants using the DNA isolation kit (Tiangen, China), according to the manufacturer's protocol. Genomic DNA was sheared into fragments and then hybridized with the xGen Exome Research Panel v1.0 probe sequence capture array from IDT (Integrated Device Technology, USA) to enrich the exonic region. The enriched libraries were analyzed on an Illumina HiSeq XTen (Illumina, USA) platform. Low-quality variations of the quality score < 20 (Q20) were filtered out. Sequencing reads were mapped to the GRCh37/Hg19 reference genome *via* Burrows-Wheeler Aligner (BWA) software. All identified variants were annotated using the 1000 Genomes Project (Chinese), dbSNP, Genome Aggregation Database (gnomAD), and ExAC database. Variants with a minor allele frequency higher than 5% were filtered out. Finally, the candidate variants were evaluated using the ACMG (American College of Medical Genetics and Genomics) criteria and further validated by direct Sanger sequencing.

### Direct Sequencing of the *CLCNKB* Gene

All the primer pairs were designed to amplify the exons of the *CLCNKB* gene (**Supplementary data Table 1**). The PCR mixture contained 1.5 μl of primers, 2.0 μl of DNA, 12.5 μl of 2 × Taq Master Mix (Vazyme Biotech Co., Ltd), 9 μl of ddH_2_O, in a total volume of 25 μl. Cycling conditions included a predenaturation step at 94°C for 5 min, followed by 34 cycles at 94°C for 30 s, 59°C for 30 s and 72°C for 30 s, with a final extension at 72°C for 5 min. The PCR products were first purified and then sequenced by BigDye Terminator (Applied Biosystems). In addition, 50 healthy unrelated controls from the Chinese population were screened by Sanger sequencing to exclude novel variants such as nondisease-associated variations. The *CLCNKB* gene variant (GenBank association number NM_000085.5) was used as a reference sequence.

### Copy Number Variation Analysis Confirmation of the Whole Gene Deletion

Using the primer pair sequences listed in **Supplementary Table 1**, copy number variation (CNV) analysis was performed. Quantitative PCR (qPCR) was performed using AceQ qPCR SYBR Green Mix (Vazyme Biotech Co., Ltd). The relative *CLCNKB* gene expression was measured by subtracting the Ct values of the three exons (E2, E10, and E20) from an endogenous control (GAPDH) gene, using the 2^-ΔΔCt^ method.

### Plasmid Construction

To create hybrid minigene constructs, we used the pSPL3 minigene reporter vector, which includes a conventional expression system with two exons (SD6 and SA2) to analyze the resultant mRNA transcripts. The minigene vector mainly produces two transcripts, one composed of exon SD6, an inserted exon, and exon SA2 (upper), and the other composed only of exon SD6 and SA2 (lower) (**Figure 2A**). To perform a minigene assay, we generated fragments containing the target exons (3, 12, and 13) where the variants were located, and 150-200 bp of flanking intronic regions with XhoI and BamHI restriction sites. These inserts were amplified by PCR from the patients' genomic DNA using primers described in **Supplementary Table 2**. Both edges of the shortened introns were properly designed by the Human Splicing Finder to avoid the activation of cryptic splicing. The pSPL3 vector was digested by restriction enzymes XhoI and BamHI, and then ligated with the purified PCR products to construct the wild-type and mutant minigene vectors using the ClonExpressTM II One Step Cloning Kit (Vazyme Biotech Co., Ltd). All constructs were confirmed by bidirectional sequencing.

### *In Vitro* Splicing Assay

HEK293 and HeLa cells were seeded in 12-well plates, with 1 mL of DMEM in each well, at 37°C in 5% CO2. When the cells were 90% confluent, cells were transfected with 1 μg pSPL3, wild-type and mutant constructs purified plasmids using Lip2000 DNA transfection reagents (Invitrogen). After 24 hours, the cell total RNA was extracted by TRIzol Reagent (Takara, Japan). The first cDNA strand was reverse-transcribed using the HiScript III RT SuperMix (Vazyme Biotech Co., Ltd). The resulting cDNA was used as a template to amplify the product, including exon 3 with the SD6 forward primer (5'-TCTGAGTCACCTGGACAACC-3') and the SA2 reverse primer (5'- ATCTCAGTGGTATTTGTGAGC-3'). RT-PCR amplification for aberrant splice transcripts, agarose gel separation, and subsequent direct Sanger sequencing were performed. Quantification of the abnormal splicing percentage was calculated as the percentage of exclusion (%) = (lower band/[lower band + upper band]) x 100. Error bars represent SEM (n=3). *P < 0.05, unpaired Student's t-test.

## Results

### Clinical Analysis

We report four cases of Batter syndrome (two females and two males). The mean age of the patients at diagnosis was 8.7 months (range, 3 m–1 y9 m). Three patients were sent to our hospital because of repeated vomiting, diarrhea, dehydration, and fever, and patient 2 because of vomiting and growth retardation. Patient 3 and patient 4 both had fevers and patient 1 presented a special face with protruding forehead.

The clinical features of our patients are listed in **Table 1**. Serum electrolytes revealed hyponatremia, hypokalemia, and hypochloremia; blood gas analysis showed metabolic alkalosis in all patients. The serum aldosterone level and the angiotensin II activity were high in all patients, and the rennin activity was activated in patients 2 and 4. No hypocalciuria was found in our patients. Electrocardiography showed that patient 1 and patient 3 had a low and flat T wave, and liver function injury was found in patient 4.

**Table 1:** Clinical and genetic analysis of four Type III Bartter syndrome patients.

		P1	P2	P3	P4
**Gender**		female	female	male	male
**Onset age**		6M	5M	1Y9M	3M
**Variants**		c.239G > A/c.1053-1G > A	c.239G > A/c.1228-2A > G	c.228A > C/Ex2_20 del	Ex2_20del/ Ex2_20 del
**Blood**					
	Na (mmol/L)	127	133	131.4	123
	K (mmol/L)	1.75	1.8	1.95	1.9
	Cl (mmol/L)	84.0	89	84.6	74.6
	Ca (mmol/L)	2.94	1.11	1.25	2.54
	Mg (mmol/L)	1.05	0.76	1.13	0.92
	PH	7.74	7.49	7.46	7.507
	HCO3 (mmol/L)	40.3	26.5	125.7	37
	PRA (ng/ml/h)	0.06	1.61	0.24	1.25
	ANG I (pg/ml)	15.62	13.73	15.91	14.36
	ANG II (pg/ml)	990.79	1313.25	864.70	1241.12
	Aldosterone (pg/ml)	195.15	174.3	153.41	183.24
**Urine**					
	Na (mmol/kg.d)	51	15.3	25	12.3
	K (mmol/kg.d)	62.8	10.6	60.14	7.37
	Cl (mmol/kg.d)	95.0	19.7	50.9	17.33
	Ca (mmol/kg.d)	2.93	0.19	0.29	0.54
	Mg (mmol/kg.d)	1.62	0.22	3.4	0.29

All patients were treated with indomethacin and salt (potassium and/or sodium) supplementations, as required. According to our follow-up, all patients showed growth retardation. Patient 2, after eleven years of follow-up and obvious growth retardation, [weight 10 kg (≤2 SD), height 79 cm (≤2 SD)] was still observed. Renal ultrasound examination showed a blurred structure in both kidney and pelvis separations in the right renal.

### Genetic Analysis

**Figure 1:**
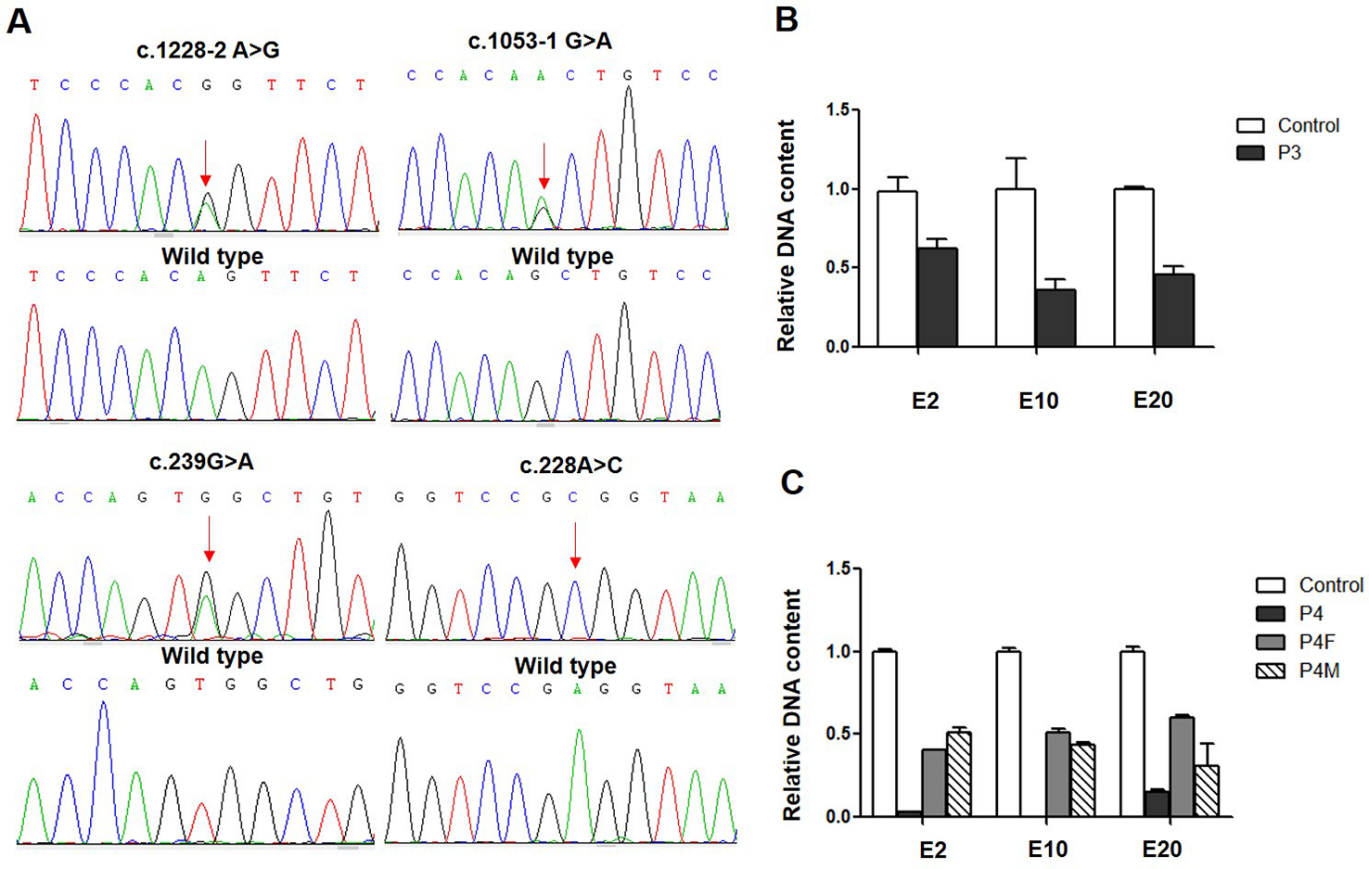
*CLCNKB* gene variants identified in type III Bartter syndrome patients. **(A)** Direct sequencing showing four point variants of the *CLCNKB* gene (arrows), the c.228G > A show only C because another allele is deleted; the wild-type sequence is also shown. **(B)** *CLCNKB* gene qPCR analysis of patient 3 showed a heterozygous loss of exons 2, 10 and 20 in *CLCNKB*. **(C)** *CLCNKB* qPCR analysis of patient 4 showed a homozygous loss of exons 2, 10 and 20 in *CLCNK*B, while both parents appeared heterozygous.

All four patients and parents underwent genetic analysis on extracted DNA from peripheral blood. By whole-exome sequencing and subsequent direct sequencing of the *CLCNKB* gene, we identified three point variants, c.239G > A, c.1053-1G > A, and c.1228-2A > G (**Figure 1A**). c.239G > A and c.1053-1 G > A were novel variants which have not been reported in the genomic databases or the literature at the time of query. The c.239G > A in exon 3 was predicted to create a stop codon at 80 (p.(Trp80*)); c.1053-1G > A was located at the -1 position of the splicing acceptor site, which may result in abnormal splice of exon 12. The c.1228-2A > G was a previous reported variant at the -2 position of the splicing acceptor site, which may result in the abnormal splice of exon 13. Patient 1 and patient 2 carried compound heterozygous variants c.[239G > A]; c. [1053-1G > A] and c.[239G > A]; c.[1228-2A > G], respectively, which were inherited from their mother and father, respectively.

Whole-exome sequencing revealed that patient 3 carried a heterozygous whole gene deletion (Ex2_20 del) (**Figure 1B**), and patient 4 had a homozygous whole gene deletion (Ex2_20 del). Copy number variant analysis by qPCR confirmed the *CLCNKB* gene exon deletion in the two patients, and patient 4 inherited the large homozygous loss of exons 2–20 (Ex2_20 del) from his parents (**Figure 1C**).

**Figure 2:**
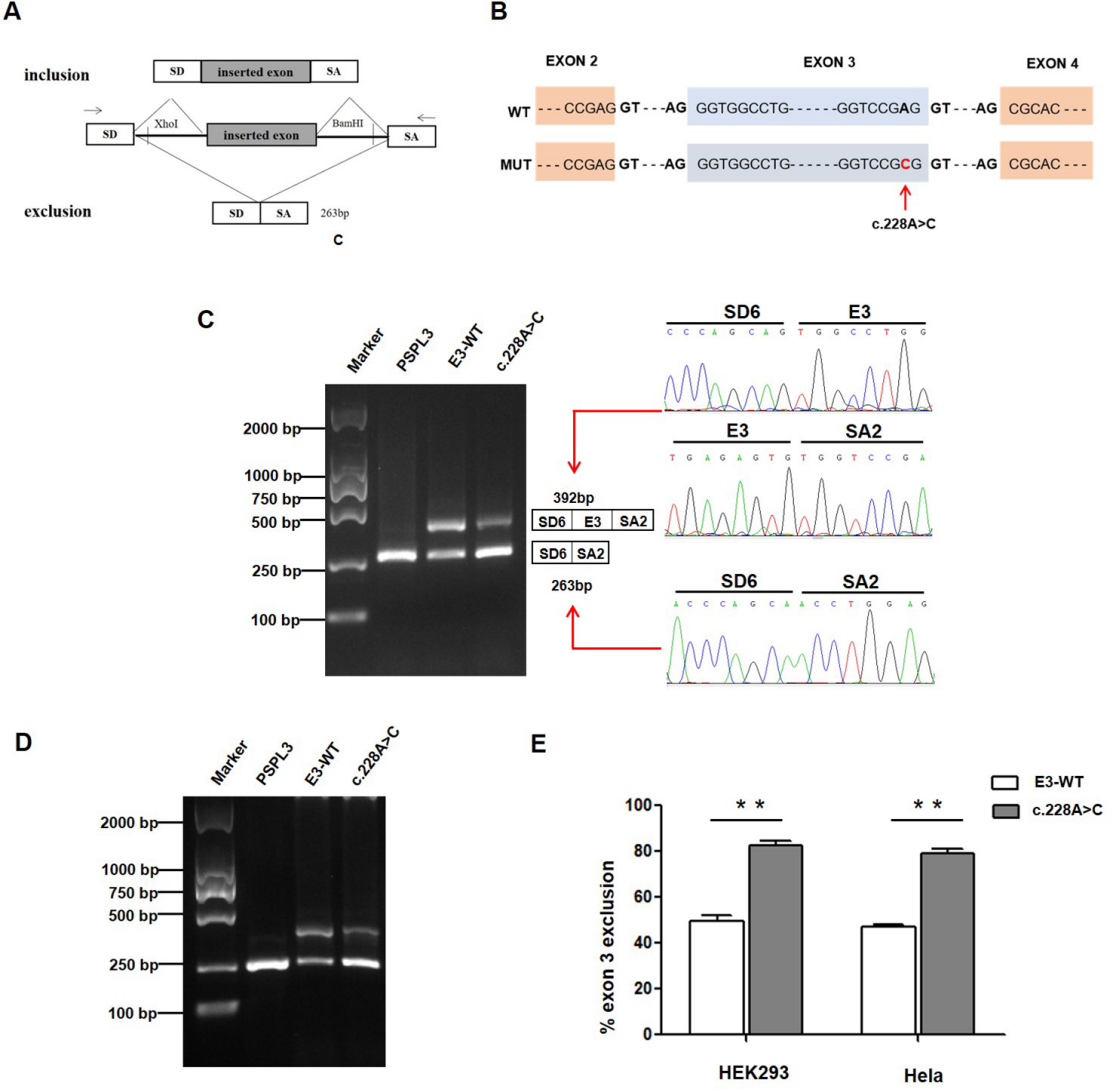
Effect of *CLCNKB* gene c.228A > C variant by Minigene assays. **(A)** RT-PCR amplified products of hybrid minigene transcripts in HEK293 cells. The transcripts produced by the hybrid minigene are schematically shown, and the arrows show the primers used to amplify (inset) (Wang et al., 2018b). **(B)** Exon 3 and adjacent structures of the *CLCNKB* gene. The arrow shows the location of the splice site variant c.228A > C in exon 3. **(C)** Gel electrophoresis of the RT-PCR product of minigene transcripts in HEK293 cell. Lane 1: marker; Lane 2: pSPL3 (263 bp); Lane 3: E3-WT (392 bp and 263 bp); Lane 4: c.228A > C (392 bp and 263 bp). The two fragments were directly sequenced (right panel). **(D)** Gel electrophoresis of the RT-PCR product of minigene transcripts in Hela cell. Lane 1: marker; Lane 2: pSPL3 (263 bp); Lane 3: E3-WT (392 bp and 263 bp); Lane 4: c.228A > C (392 bp and 263 bp). **(E)** Quantification of the splicing percentage in HEK293 and Hela cells was densitometrically calculated on a molar basis as the percentage of exclusion (%) = (lower band/[lower band + upper band]) x 100. Error bars represent SEM (n=3). **P < 0.01, unpaired Student's t-test.

In patient 3, we also identified a heterozygous synonymous variant c.228A > C (p.(Arg76Arg)) located at -2 bp of the splice donor site in exon 3 (**Figure 2B**). This variant has not been reported in the genomic databases and was not found in 50 matched healthy controls. The Human Splicing Finder prediction suggested that c.228A > C may alter the WT donor site (decreasing the score from 76.76 to 47.81); the BDGP prediction showed that c.228G > A decreased the donor score from 0.95 to 0.84.

### Splicing Minigene Reporter Assay

To verify whether the c.228A > C (p.(Arg76Arg)) variant affected mRNA splicing, we next performed a minigene splicing assay *in vitro*. After the minigene plasmids with the inserted c.228A > C fragment were transfected into HEK293 and HeLa cells, total RNA was extracted and transcribed to cDNA. RT-PCR was performed using flanking primers and then visualized on an agarose gel (**Figure 2C**). As a result, two fragments were uniquely detected from the RT-PCR products of the E3-WT and c.228A > C in double cells. The product sequencing revealed that the larger amplicon of 392 bp was the exon 3–included transcript, while the smaller splice of 263 bp was the exon 3–excluded transcript (**Figures 2C, D**). The amount of exon 3-skipping transcripts of c.228A > C were significantly increased compared with E3-WT (47% versus 80.2% in HEK293 and 45% versus 79.3% in Hela cells, respectively) (**Figure 2E**).

**Figure 3:**
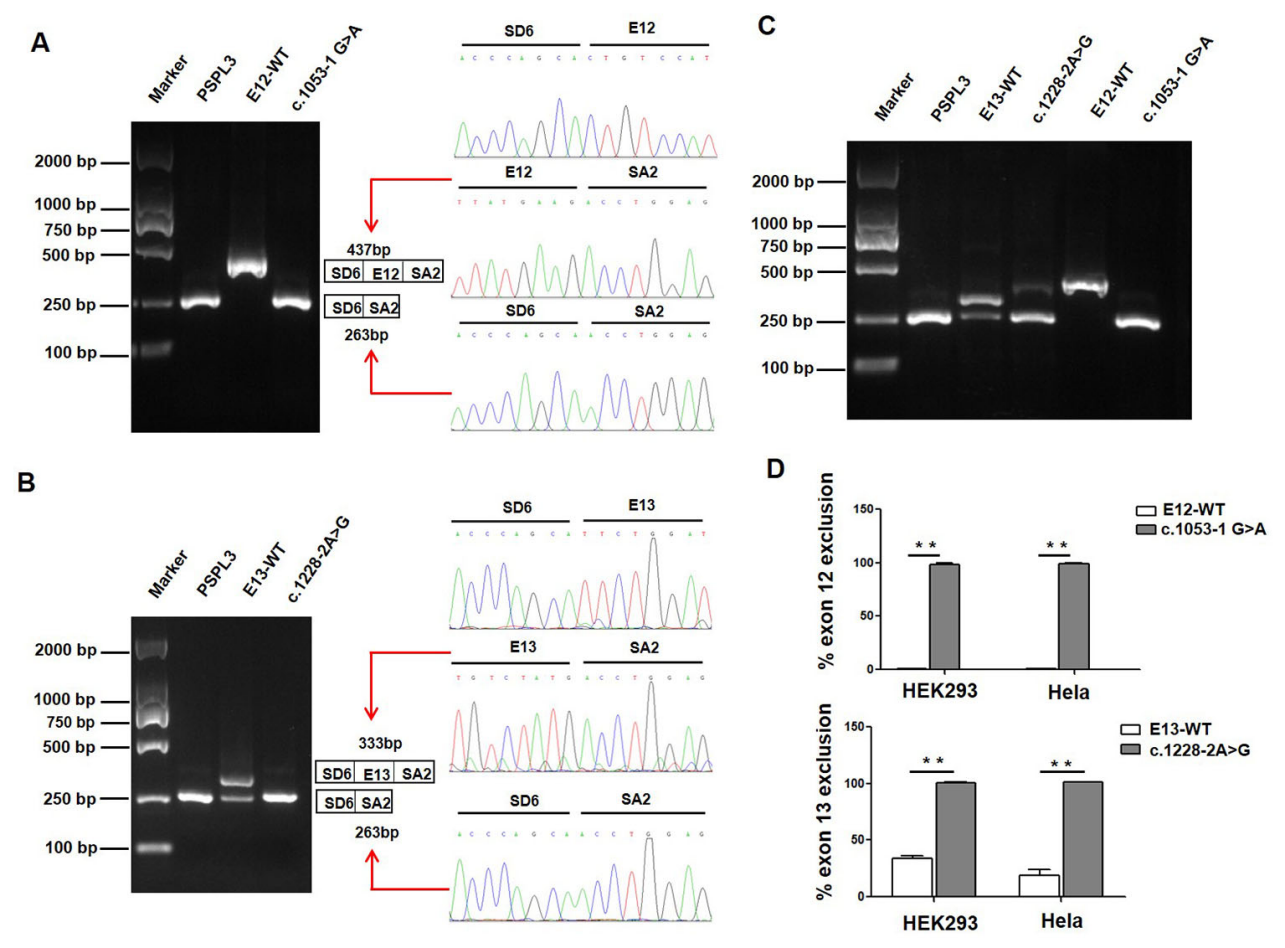
Effect of *CLCNKB* gene c.1053-1G > A and c.1228-2A > G variants by Minigene assays. **(A)** Gel electrophoresis of the RT-PCR product of c.1053-1G > A transcripts in HEK293 cell. Lane 1: marker; Lane 2: pSPL3 (263 bp); Lane 3: E12-WT (437bp); Lane 4: c.1053-1 G > A (263 bp). The two fragments were directly sequenced (right panel). **(B)** Gel electrophoresis of the RT-PCR product of c.1228-2A > G transcripts in HEK293 cell. Lane 1: marker; Lane 2: pSPL3 (263 bp); Lane 3: E13-WT (333 bp and 263 bp); Lane 4: c.1228-2 A > G (263 bp). The two fragments were directly sequenced (right panel). **(C)** Gel electrophoresis of the RT-PCR product of minigene transcripts in Hela cell. **(D)** Quantification of the splicing percentage in HEK293 and Hela cells was densitometrically calculated on a molar basis as the percentage of exclusion (%) = (lower band/[lower band + upper band]) x 100. Error bars represent SEM (n=3). **P < 0.01, unpaired Student's t-test.

We also evaluated the effect of the c.1053-1 G > A and c.1228-2 A > G variants on splicing using the pSPL3 minigene reporter in HEK293 and Hela cells. Analysis of cDNA prepared from HEK293 and Hela cells revealed that the c.1053-1G > A and c.1228-2A > G variants produce the complete skipping of exon 12 and exon 13, respectively (**Figure 3**), indicating that both splice site variants disturbed the normal splicing *in vitro*.

## Discussion

In this study, we identified four patients who had compound heterozygous variants or homozygous variants of the *CLCNKB* gene, including a novel nonsense variant c.239G > A (p.(Trp80*)), two splice site variants (c.1053-1G > A and c.1228-2A > G), a whole gene deletion and a novel synonymous variant c.228A > C. The clinical features present in our four patients are generally consistent with the pathophysiology found in Bartter syndrome, including hyponatremia, hypokalemia, hypochloremia, repeated vomiting and growth retardation. By gene analysis, all four patients were diagnosed with type III BS.

In the clinic, type III BS manifests highly variable phenotypes, ranging from an early-onset and severe antenatal BS to a late-onset and mild Gitelman's syndrome (GS) (Zelikovic et al., 2003; Fukuyama et al., 2004; Gorgojo et al., 2006; Tajima et al., 2006). The functional severity of the mutant channel has been proposed to explain this phenomenon. *Cheng* et al. established a genotype-phenotype association and revealed that the functional severity of *CLCNKB* genotypes correlated with age at onset, plasma chloride concentration, and urine calcium excretion rate (Cheng et al., 2017). Keck et al. also reported that *CLCNKB* mutations with milder functional outcomes were linked to older age at diagnosis of classic BS (Keck et al., 2013). In this study, patient 1 and patient 2 both carried compound heterozygous variants c.[239G > A]; c.[1053-1G > A] and c.[239G > A]; c.[1228-2A > G], respectively. Our minigene assay suggested that c.1053-1G > A caused exon 12 skipping, which keeps the reading frame, and would produce the loss of 58 amino acids (codons 352-409). This region contained a part of a highly conserved D8 transmembrane domain, which many variants have been reported to be involved in with regards to the impaired CIC-Kb function (Keck et al., 2013). Likewise, c.1228-2A > G induces exon 13 skipping, which introduces a PTC 46 codons downstream (F410Efs*46), which would truncate the protein and subsequently lose the C-terminal region that would compromise CIC-Kb function. The two patients were diagnosed early in their infancy with severe hyponatremia, hypokalemia, and hypochloremia, supporting the hypothesis that truncating variants of *CLCNKB* may be correlated with a severe phenotype in type III BS patients.

Patient 4, whose onset age was 3 months with more severe dehydration, electrolyte imbalance, vomiting, and growth retardation, was homozygous for the whole *CLCNKB* gene deletion. *Shao* et al. reported that deletion of the complete *CLCNKB* gene was the most common variant in Chinese patients with cBS, and the frequency of whole gene deletion was up to 9/28 (32%). Patients who carried the whole *CLCNKB* gene deletion variant showed an early-onset, severe phenotype with greater urinary salt wasting (Han et al., 2017; Seys et al., 2017; Li et al., 2019). Taken together, our results support that the whole *CLCNKB* gene deletion may account for a more severe phenotype of patients.

In patient 3, we identified a novel synonymous variation c.228A > C (p.(Arg76Arg)), in addition to a heterozygous whole *CLCNKB* gene deletion. *In silico* analysis suggested that this synonymous variation may affect the WT donor splice site. Our minigene assay in HEK293 and Hela cells both showed that the c.228A > C change disturbed normal splicing by increasing ~30% exon 3 exclusion compared with WT. Interestingly, in the case of the exon 3 minigene, we observed that ~45% of transcripts of the wild type minigene do not include exon 3. In fact, exon 3 is alternatively spliced in physiological conditions. This similar alternative splicing was also reported in several large multiexon minigenes of the *BRCA2* gene (Acedo et al., 2015; Fraile-Bethencourt et al., 2017). The *CLCNKB* gene has a natural transcript (NM_001165945, ENST00000375667.7) in which exon 3 is excluded, producing a smaller isoform (517aa). Thus, in the *in vitro* pSPl3 minigene assay, E3-WT also detected exon3 skipping transcript from the RT-PCR products. Given that this patient carries a whole *CLCNKB* gene deletion in trans-allele, the low amount (20%) of the full-length transcript produced by the c.228A > C allele is not enough to keep ClC-Kb activity. Taken together, we believe that the compound heterozygous variants (c.228A > C and whole *CLCNKB* deletion) are the molecular basis of this BS patient. In our previous study, we identified that a synonymous variant c.1755A > G (p.(Thr585T)) in a type III BS patient located in exon 15 resulted in abnormal mRNA splicing and a subsequent defect in the chloride transport function of ClC-Kb (Wang et al., 2018).

## Conclusion

In conclusion, we reported five *CLCNKB* gene variants leading to type III BS in four patients. Our results support that truncating and whole gene deletion variants of the *CLCNKB* gene correlated with the severe phenotype of type III BS patients. Notably, we identified that the synonymous variant c.228A > C and two classical splice site variants (c.1053-1G > A and c.1228-2A > G) disturbed the normal mRNA splicing *in vitro* and subsequently caused type III BS. Our study suggests that a significant portion of synonymous substitutions and splice site variants play an important role in the molecular basis of type III BS, and careful molecular profiling of patients will be essential for future effective personalized treatment options.

## Data Availability Statement

All datasets generated for this study are included in the article/**Supplementary Material**.

## Ethics Statement

The study was performed according to the ethics committee of the Children's Hospital of Nanjing Medical University (Nanjing, China). Written informed consent was obtained from all the patients and their parents.

## Author Contributions

AZ, SH, ZJ, GD, and FZ conceived and designed this study. CW and YH wrote the manuscript and performed the experiments. JZ collected the clinical samples and clinical data. YH wrote the clinical part of the manuscript. BZ and WZ performed NGS analysis. HB, GD, and FZ reviewed and edited the manuscript.

## Funding

This work was supported by the National Natural Science Foundation of China (Nos. 81800589, 81800652) and the Science and Technology Development Foundation of Nanjing Medical University (Nos. 2017NJMUZD05).

## Conflict of Interest

The authors declare that the research was conducted in the absence of any commercial or financial relationships that could be construed as a potential conflict of interest.
