# Supplementary material for: Splicing Characterization of CLCNKB Variants in Four Patients With Type III Bartter Syndrome
Source: Front Genet. 2020 Feb 21;11:81. doi: 10.3389/fgene.2020.00081 (PMC7047732; doi:10.3389/fgene.2020.00081)
Supplement: Supplementary file 1 [file DataSheet_1.docx]

**Supplementary Table 1. Primers used for amplification and sequencing of the coding regions of the *CLCNKB* gene.**

| ***CLCNKB* region** | **Forward primer (5′→3′)** | **Reverse primer (5 ′→3 ′ )** |
| --- | --- | --- |
| Exon 2 | ACTGGAAGGGCCTAGAGGCAGT | GATGTCCTGAGTGGTCCTCCAG |
| Exon 3 | TGCCCCACCCTGTGCCGTGAC | CTTGGCCCAGAGCAGCACCTG |
| Exon 4 | TAAACCTGGGATATGGACCCA | AACAGGCTTCGCAGAAGATCC |
| Exon 5-7 | GATCTGGCGAGATCGTAATG | TACACTGAAGCCTCTTAGCAC |
| Exon 8 | ATTCCAGGCTGGACGGGTCT | ACTTGTAGGTGGTGTTAGGG |
| Exon 9-10 | TGGAATGCCAGGACACAGATT | GTATAGACTCCAGGAGATATGG |
| Exon 11-12 | ATTCTCCTATCAGCCTCTACCC | TTGTCCAGGACCATGCAATGG |
| Exon 13-14 | GGTGTTTACTGGGAAGGCTAA | GAGGACTCAGTGACTGCTCCG |
| Exon 15 | CATCACTCCCTCGTGGCTCCTG | CTACGGTGGCGTTTCTTTTTCG |
| Exon 16 | AATTCGCAACTGGGTCATCGG | AAGGTCACACGGTTGGTTGCT |
| Exon 17-18 | TGCCTCTGAGCCAAAGTTT | AACAGGGCAGGAGCTTCTT |
| Exon 19 | CGACTTAACCACTCAGGACAGA | AAGAAGAGGGACTCATC |
| Exon 20 | TGGCCAGTGGCCAGCCTGCC | TGGGCTAGGTTAAAGTTGCC |

| **Nucleotide change** |  | **Primers (5’-3’)** | **Tm** |
| --- | --- | --- | --- |
| c.228G>A | XhoI-E3F | atcaccagatatctgggatccACTGGAAGGGCCTAGAGGCA | 61 |
|  | BamHI-E3R | accagaattctggagctcgagGATGTCCTGAGTGGTCCTCCAG | 60 |
| c.1053-1G>A | XhoI-E12F | accagaattctggagctcgagTAGATGATACTACAGCTTCGGGAGG | 61 |
|  | BamHI-E12R | atcaccagatatctgggatccCTGCCAGAGTCTGGCATTAGGG | 63 |
| c.1228-2A>G | XhoI-E13F | atcaccagatatctgggatccTAATGCCAGACTCTGGCAGTGG | 62 |
|  | BamHI-E13R | accagaattctggagctcgagGAGGACTCAGTGACTGCTCCGT | 60 |

**Supplementary Table 2. Primer sequences for minigene fragments in the *CLCNKB* gene.**
